# Protocol for a Multi-Level Policy Analysis of Non-Communicable Disease Determinants of Diet and Physical Activity: Implications for Low- and Middle-Income Countries in Africa and the Caribbean

**DOI:** 10.3390/ijerph182413061

**Published:** 2021-12-10

**Authors:** Maylene Shung-King, Amy Weimann, Nicole McCreedy, Lambed Tatah, Clarisse Mapa-Tassou, Trish Muzenda, Ishtar Govia, Vincent Were, Tolu Oni

**Affiliations:** 1 Health Policy and Systems Division, School of Public Health and Family Medicine, University of Cape Town, Cape Town 7925, South Africa; MCCNIC003@myuct.ac.za; 2 African Centre for Cities, University of Cape Town, Cape Town 7701, South Africa; amy.weimann@uct.ac.za; 3 Research Initiative for Cities Health and Equity (RICHE), Division of Public Health Medicine, School of Public Health and Family Medicine, University of Cape Town, Cape Town 7925, South Africa; trishmuzenda@gmail.com (T.M.); Tolullah.Oni@mrc-epid.cam.ac.uk (T.O.); 4 Health of Populations in Transition Research Group (HoPiT), University of Yaoundé I, Yaoundé 8046, Cameroon; Lambed.Tatah@mrc-epid.cam.ac.uk (L.T.); mapatassou@yahoo.fr (C.M.-T.); 5 Global Diet and Physical Activity Research Group, Medical Research Council Epidemiology Unit, University of Cambridge, Cambridge CB2 0QQ, UK; 6 Caribbean Institute for Health Research, The University of the West Indies, Mona Kingston 7, Jamaica; ishtargovia@gmail.com; 7 Center for Global Health Research, Kenya Medical Research Institute (KEMRI), P.O. Box 1578, Kisumu 40100, Kenya; vincentwere@gmail.com

**Keywords:** multi-level policy analysis, noncommunicable diseases, policy analysis, diet, nutrition, intersectoral, low- and middle-income countries

## Abstract

Non-communicable diseases (NCDs) are the leading cause of death globally. Despite significant global policy development for addressing NCDs, the extent to which global policies find expression in low-and-middle income countries’ (LMIC) policies, designed to mitigate against NCDs, is unclear. This protocol is part of a portfolio of projects within the Global Diet and Activity Research (GDAR) Network, which aims to support the prevention of NCDs in LMICs, with a specific focus on Kenya, Cameroon, South Africa and Jamaica. This paper outlines the protocol for a study that seeks to explore the current policy environment in relation to the reduction of key factors influencing the growing epidemic of NCDs. The study proposes to examine policies at the global, regional and country level, related to the reduction of sugar and salt intake, and the promotion of physical activity (as one dimension of healthy placemaking). The overall study will comprise several sub-studies conducted at a global, regional and country level in Cameroon, Kenya and South Africa. In combination with evidence generated from other GDAR workstreams, results from the policy analyses will contribute to identifying opportunities for action in the reduction of NCDs in LMICs.

## 1. Introduction

Non-communicable diseases (NCDs) are the leading cause of death globally [1,2,3]. Unlike previous assumptions that NCDs are mainly a disease-burden in high-income country (HIC) contexts and linked to affluence, the prevalence of NCDs is rising significantly in low- and middle-income countries (LMICs) and in poor urban populations of HICs [4]. NCDs account for 78% of all deaths in the Caribbean, for example, and is projected to become the leading cause of deaths in Africa by 2030 [5].

The rising levels of NCDs have many underlying factors, necessitating a multi-faceted approach to their prevention and reduction [4]. A widely acknowledged contributory factor is the rapid urbanization of populations. In LMICs, for example, NCD-prevalence has risen in both wealthy and poor urban populations [6]. The rising NCD burden, associated with rapid urbanization, is characterized by significant nutritional and physical activity transitions, especially as the urban poor’s dietary patterns and living conditions change and obesogenic environments (meaning environments that promote overweight and obesity) predominate [7]. These nutritional transitions are characterized by a shift in diets away from traditional foods, towards easily accessible fast-foods and supermarket products high in refined sugars, salt, saturated fats and carbohydrates [4]. The Physical activity (PA) transition is characterized by the increased use of technologies and methods designed to make work and travel more efficient and less reliant upon physical activity [8]. These obesogenic environments present significant risk factors for many NCDs, including diabetes and hypertension [9]. At a global level, the prevalence of NCDs has been underscored by Sustainable Development Goal (SDG) 3, target 3.4, which calls for ‘the reduction by one-third of premature mortality from NCDs through prevention and treatment and the promotion of mental health and wellbeing’ [10]. Several global policy documents endorsed by United Nations (UN) member states contain recommendations for policy actions at national and subnational levels within member states.

Despite the increasing availability of NCD global policies and the expressed commitment of signatory countries to the global declarations and targets for NCD prevention and control, the implementation of concerted actions in settings where the NCD burden is high and/or rapidly increasing has been limited. The World Health Organization’s (WHO) systematic review on NCD control strategies between 1995–2006 and the ensuing report, “Interventions on Diet and Physical Activity: What Works”, suggested interventions in eight categories: policy and environment; mass media; school settings; the workplace; the community; primary health care; older adults; and religious settings [11]. However, there are significant gaps in knowledge about NCDs and what the most effective intersectoral strategies for policy development and implementation at national and subnational levels are, particularly in LMICs.

Similarly, there is scarce knowledge on the extent to which global NCD-related policies find expression in, or have relevance to, LMICs. A 2013 review found a limited policy uptake of global policies in LMICs, as evidenced by relatively few national NCD-related policy documents identified by the authors, despite conducting an extensive search [12]. Similarly, a systematic review conducted on the evaluation of the ‘WHO best buys’ in LMICs for the period of 1990–2015 found little evidence pertaining to the implementation and effect of these WHO recommendations in LMICs [13]. The majority of studies were from India and most focused on tobacco and cancer, with research evaluating diet and physical activity as significant contributors to NCD prevalence being scarce. Limited research on the determinants of NCDs is available in other LMIC regions. A more recent 2018 review, which examined ‘best-buys’-related policies in five African countries, is one of a handful of empirical studies relating to LMICs in this region [14].

This study forms part of a portfolio of projects of the Global Diet and Activity Research (GDAR) Network. The GDAR [7] has the overall goal of contributing to the prevention of NCDs through generating knowledge and exploring potential interventions on various facets of NCDs as experienced in LMICs. Funded through a UK National Institute of Health Research grant, the GDAR Network is comprised of institutions in the UK, Africa and the Caribbean. This study relates to the policy workstream of GDAR, aimed at exploring policies at different levels of the global system, and, in particular, the extent to which global policies have relevance for, and find expression in, LMICs, particularly in the Africa region, whilst drawing on lessons from, and for, the Caribbean. This study is based on the premise that global policy drivers in the NCD space influence policy ideas and intentions at other levels of the global system, such as at regional and country levels. We further postulate that national-level policies may in turn shape policy intentions and practices at subnational (local) levels, but equally recognize that independent local innovations may occur. At a subnational level, cities are increasingly recognized as important localities of significant policy change and implementation, given the global urbanization phenomenon.

This study focuses on analyzing policies related to diet and physical activity as two key NCD determinants. For diet-related policies, we will focus on those with implications for sugar and salt intake reduction, and then on policies that address physical activity. From the published policy reviews in the five African countries, most existing diet-linked policies focus on these two nutrients. Furthermore, sugar reduction is a particular focus in children and adolescents. The concept of healthy “placemaking” (sourced at https://www.pps.org/article/what-is-placemaking on 18 December 2018), refers to the convergence of civil society agency, with government and private sector actors, organizations and structures, in creating environments that are conducive to healthy living and being, and that consider all facets of life in a community, has attracted a significant level of global attention. Our investigation of healthy urban spaces in GDAR adopted this concept to understand the extent to which policies support environments that facilitate the availability of healthy, affordable food, and safe and pleasant spaces for physical activity.

While there exists a growing body of research on the subject of NCDs and their determinants, there is relatively sparse knowledge on the role and influence of the policy environment at global, regional and country levels and the extent to which these policies address important determinants of diet and healthy placemaking. Additionally, the extent to which current global and regional policies on NCDs find expression in country-level policies, is uncertain.

By conducting a detailed analysis of the way policies at multiple levels address these aspects, we hope to provide insight for decision-makers across relevant sectors. Given the scope and focus of GDAR, we pay special attention to policy proposals aimed at adolescents, whilst also scrutinizing gender considerations.

This paper outlines the protocol for the study, which aims to explore the current policy environment for diet and physical activity-related NCD policies at multiple levels.

## 2. Study Aim and Conceptual Frameworks

The overall aim of this study is to explore whether (and which) global and regional policy interventions are promoted for the reduction of NCDs (focusing on good diet and physical activity) in LMICs, particularly in Africa, and how these policy proposals find expression at the country level.

**Figure 1:**
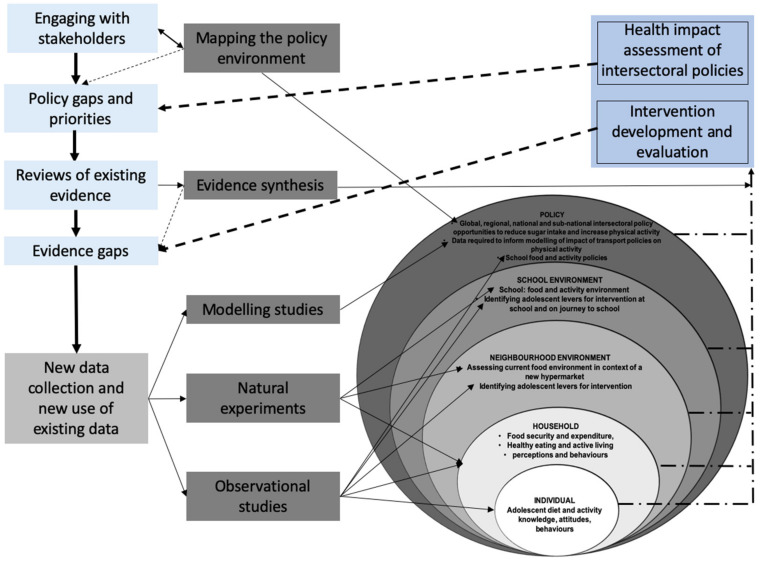
Conceptual framework for policy-relevant impactful research in the GDAR network-with permission [7].

As an important factor that influences NCDs, the role and place of policy are outlined in the ecological framework in Figure 1 to illustrate that the factors influencing dietary intake extend well beyond individual choice and behavior, to a multitude of systemic and structural factors. The ecological framework suggests that complex factors influencing diet span across multiple sectors and are nested within and interact with broader drivers [15]. This highlights that multi-level interventions for the mitigation of NCDs are not the domain of the health sector alone, but require coordinated intersectoral actions across government and society [16]. This also holds true for other NCD risk factors such as physical activity.

Policies and laws constitute an important layer of influence contributing to the creation of a structural and systemic environment that may be health-promoting or harmful to citizens. Policies operate at many different levels, including global, regional, national and subnational levels. This includes policies that operate at the community and facility level at the subnational level, such as a school-level policy regarding tuck shop goods.

As suggested in the ecological framework approach, depicting barriers and opportunities for healthy eating by Afshin et al. [17] and Mozaffarian et al. [18], determinants of NCDs traverse multiple layers—from the individual, sociocultural, community, through to governmental and global. In the governmental and global sphere, government and global policies are considered important determinants in shaping the food and physical activity landscape of a country and the communities within a country, as depicted in Diagram 1, which outlines the approach of the GDAR network portfolio of research [7]. Figure 1 indicates how this study interacts with the other research streams in the overarching GDAR research portfolio and the way an understanding of the policy landscape will allow for a broader understanding of the way NCD determinants and possible interventions could work together in creating context-appropriate interventions to mitigate NCDs. Furthermore, the nested layers, akin to the ecological approach, demonstrate the role and place of policy in the multiple layers that shape the environment for NCD-related determinants and interventions.

In this study, we focus on diet and physical activity as two crucial determinants for preventing and reducing NCD prevalence. Given the strong global influence of factors such as healthy food availability, we will focus on policies that operate at a global and regional level and explore how these policies influence national and subnational policy design and content. We will also explore the current policy environment of the different sectors that perform a role in the prevention and reduction of diet- and activity-related NCDs, which is strongly dependent on addressing the multiple determinants of health as depicted in Figure 1. We will explore the policy environment through a policy document analysis. A document analysis (also referred to as document review) is a commonly applied research method [19]. In the field of health, a policy analysis is used to explore different aspects of policy, either prospectively in the analysis ‘*for*’ policy, or retrospectively in the analysis of policy [20]. Whilst often used in tandem with primary data collection methods, it can be used as a credible ‘stand-alone’ method, depending on the study question. As this study comprises one part of the portfolio of GDAR projects, it complements the primary studies in GDAR that focus on understanding the detailed lived experiences of adolescents and their families, and the practices of intersectoral service providers, policymakers and industry in different country contexts.

**Figure 2:**
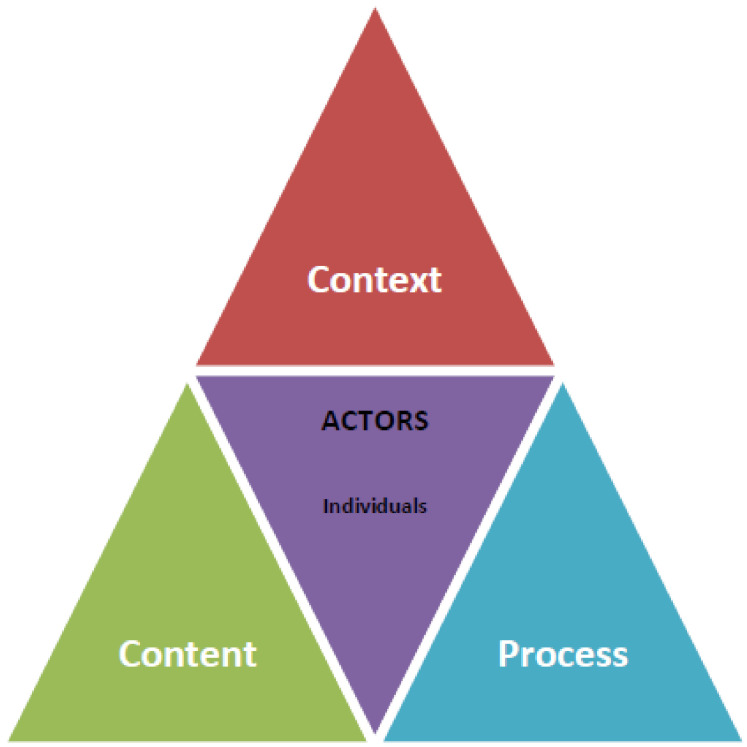
Dimensions of the Policy process-Walt and Gilson Policy Triangle. Source: Walt and Gilson [22]—with permission.

A commonly applied policy analysis framework in LMIC policy analyses, whether employing primary or secondary data collection means, is the policy triangle of Walt and Gilson [21,22]. The triangle illustrates four dimensions (Figure 2), and the interlinkages between them, for consideration when conducting a policy analysis, namely, the context in which policy is made; the process by which the policy is placed on the agenda and then developed; the actors who are fundamental in shaping policy development and implementation; and the substantive content of the policy proposals.

Further to the four dimensions of the policy triangle, the Institute for Development studies at the University of Sussex offers a policy analysis framework that highlights policy narrative and discourse, and politics and interests of the variety of policy actors as important additional influences to the way policy is shaped [23]. Having applied this framework in various research endeavours, particularly in sub-Saharan Africa, the authors highlight that “it [the research] has also focused on how ‘global’ debates play out in ‘local’ policy contexts, and how, in return, local activities are incorporated into global networks and encompass a cast of characters.”, underpinning the importance of understanding the integral linkages between global and local policy and the factors that shape these policy endeavours. This study will therefore engage, in particular, with the narratives outlined in the content of the identified policy documents, and look for instances where policy documents reflect the politics and interest of the policy actors who engaged in the various policy development processes.

Using these frameworks as a guide to our methodology, data gathering and analysis, we seek to explore the following research question: What are the policy factors that shape the prospects for promoting reduced sugar and salt intake and healthy placemaking (built environment) towards reduction of NCDs in LMICs in Africa and the Caribbean?

The overall study objectives are outlined below, with each of the separate sub-study enquiries having additional context-specific objectives, which will be addressed in the subsequent studies and papers.

1. To conduct a scoping review (between 2000–2019) of global, regional, national and local (city) policies (declarations, policy statements, written statement, policy intent) to identify the key policy opportunities for intersectoral action (earlier sentinel documents were included, as appropriate).
2. To conduct a policy content analysis on policies that meet the inclusion criteria, across relevant sectors, identified from the scoping review to identify the key policy proposals for addressing diet and physical activity interventions.
3. Examine the implications of global and regional policy prescripts for African LMICs.
4. To examine whether and how policy is transferred from the global to the local level, and how global and regional policies find expression at a country level.
5. To explore whether and how policy prescripts provide for intersectoral action in the mitigation of NCDs.
6. Lastly, explore how policies address adolescents as an important subpopulation in the prevention of NCDs, and to highlight gender as an important construct in diet and physical activity-related NCD interventions.

## 3. Study Settings

The study comprises seven ‘sub-studies’, which will be conducted at different levels of the global policy system. At each level, we will focus on NCD policies related to diet and physical activity. The foci of the seven sub-studies will differ slightly and are as follows: two sub-studies on global level policies (one on diet and one on physical activity); one on global and regional level policies, with a focus on the role of intersectorality; two at the country level, with a focus on diet and physical activity policies and how country-level policies engage with global policy proposals (Cameroon—two separate studies for diet and physical activity policies; and Kenya—the study will combine diet and physical activity policies); and a final sub-study on policy transfer of sugar reduction policies across multiple levels of the global system, with a focus on how higher-level policies found expression at country level (South Africa).

We base the approach to our study on a number of premises. Firstly, we understand that policy has many complex and inter-linking dimensions and is concerned with more than that which is expressed in written policy statements and documents. However, our study will exclusively engage in a document analysis and will primarily work with publicly available, official, written, policy documents and statements. As policy documents generally have limited details on the context within, and the process through which, policy is developed, we will primarily focus on the substantive content of the policy proposals. We will, however, extract any available information about the policy context, the actors and/or actor networks that are explicitly and implicitly referred to in the policy, as well as describe, where possible, the process through which the policy was developed and designed. We will focus on analyzing the narratives present in the policy documents using a discourse-analysis approach. Where evident, we will highlight manifestations of political interest and power in policies.

We adopt an expanded definition of policy and will include all available policy statements, declarations, policy guidelines and policy proposals. Unofficial position statements, research papers, policy briefs, and implementation guides, of which there are numerous, will be archived for further use in the GDAR network. Depending on the nature of the document, we may include final drafts of policy documents that only require final legitimation.

Whilst implementation is a critical aspect of policy, we will only focus on implementation as expressed in policy documents. We endeavor to explore implementation imperatives in the next phase of the GDAR work, through engagement with country-level policymakers and practitioners.

Given the multiple sectors implicated in diet and placemaking policies, we will source policies from purposively identified sectors. A preliminary list of key sectors has been developed through discussion within the research team and in consultation with some researchers from the wider GDAR network, drawing on our collective insights and experiences.

Within the overall conceptual frameworks and study premises, additional conceptual frameworks and theories may be used in the sub-studies to guide their enquiry, where deemed necessary.

## 4. Detail of Study Methods

### 4.1. The Scoping Reviews

Small teams of researchers from the research group will conduct scoping reviews of global, regional, national and local (city) policies (declarations, policy statements, written statement, policy intent), released between 2000–2019. The scoping review research will seek to identify the key policy opportunities for the reduction of sugar and salt intake (as two key diet-related interventions which we will focus on in this phase of the GDAR work) and the promotion of physical activity as part of healthy placemaking. The year 2000 coincides with the commencement of the 15-year Millennium Development Goal period and the year in which NCDs began to gain traction on the global agenda. Earlier sentinel documents will be referenced where relevant.

At global, regional and national levels, the scoping reviews (desktop research) aim to:

- Identify policies across different sectors with implications for diet (reduction of sugar and salt intake specifically) and physical activity.
- Identify key characteristics of each policy, and the extent to which they incorporate an intersectoral approach to sugar and salt reduction and healthy placemaking.
- Examine the ways through which policies have the potential to impact on the reduction of sugar and salt intake and the promotion of healthy placemaking.

At a country level, where policy documents might be difficult to find or identify, key informant engagements will be drawn upon to identify and obtain relevant documents.

The following search terms will be used in various combinations to guide the scoping review and to identify available policies related to NCDs, diet and physical activity via the PAIS Index and the Sabinet Legal database, and via the PubMed, Google Scholar and Science Direct search engines: non-communicable disease(s) (and noncommunicable disease(s) and NCD(s) as alternative spellings), chronic disease(s), diet, nutrition, food, sugar(s), glucose, sugar-sweetened, salt, sodium, physical activity, physical inactivity, sedentary, physical education, exercise, sport, walking, cycling, public transport, built environment, and urban planning. Organisational websites will be hand-search as required.

We will chart the evolution of past and current WHO and other UN agencies intersectoral policy/guidance documents for NCDs, with a focus on policies specifically aimed at, or that have implications for, the reduction of sugar and salt intake and the promotion of physical activity.

### 4.2. Identifying Key Sectors for Purposive Policy Document Identification

The research team discussed and developed consensus on a list of preliminary sectors of specific relevance in diet and physical activity-related policies. For diet, key sectors comprise health, food and agriculture, urban development, youth affairs, and education. The key sectors identified for physical activity are health, transport, sports and recreation, education, youth affairs, and urban development. These sectors differ slightly at the global, regional and country-level, and the sector-specific search will be adjusted accordingly for each sub-study. Other relevant sectors will be identified as they emerge during the scoping review.

Following this, the relevant policy-making agencies and organizations were then identified for each of these sectors, at each level. For example, at the global level, the websites of the following diet-related agencies/organizations will be hand-searched for relevant policy documents: the WHO, the Food and Agriculture Organization, the World Food Programme, UN-Habitat, the World Bank, UNICEF, UN Healthy Cities, UN System Standing Committee on Nutrition, World Obesity, and the World Health Assembly.

In cases where copies of the policy documents are difficult to obtain, we will supplement the desktop retrieval of policies to secure a copy by contacting other researchers and colleagues who work in government services and/or relevant international agencies and organizations, such as WHO Geneva, UN-HABITAT, and WHO-AFRO, or country-level ministries for example. The sectors to be explored will vary slightly at the country level, as countries have different ways in which they cluster their government activities.

At the country level in Kenya and Cameroon, key informants will help to identify, and retrieve, additional policy documents that are not available on websites. Supplementary material, File S1 contains a template letter to be used when requesting assistance to access policy documents.

Documents are subjected to the following inclusion and exclusion criteria:

Documents will be considered for inclusion if they are written by an actor, agency, or association that provides a global, regional, or national-level position related to the topic of NCDs and/or diet (including related concepts such as nutrition, salt, and sugar), and/or physical activity. Documents will be excluded if they have neither an explicit link (determined through document titles) or implicit link (identified through internal document keyword searches) to NCDs, diet or physical activity. Supplementary documents such as policy briefs, reports, technical notes, and best practices, will be excluded from the analysis, but retained for background information. The inclusion and exclusion process will be conducted by a research team.

**Figure 3:**
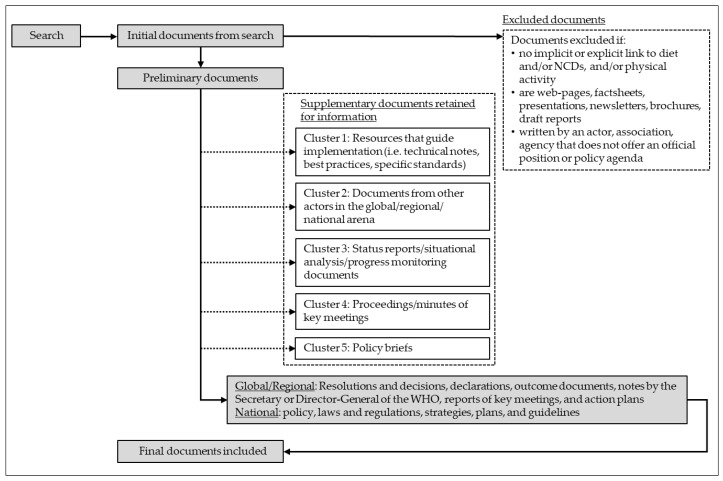
Flow diagram of proposed data cleaning and document clustering process for the global, regional, and country level policy analysis.

The results of the search will be presented as outlined in Figure 3.

#### 4.2.1. Data Storage, Coding and Analysis

Selected documents will be archived in a reference manager, such as Mendeley. Documents will be clustered in different categories and the included documents will be archived in a separate folder. Other documents that will not be included in the policy analysis will be archived for use in the broader GDAR network for various purposes.

Qualitative data analysis software, such as NVivo 12, will be used to develop a codebook (adapted to each level of analysis), data coding, extraction and analysis. We provide a template with suggested themes to guide the development of a codebook for each of the sub-studies (Supplementary material, Table S1). We will combine a thematic and interpretive content analysis of the identified policies. The deductive framework for the thematic analysis will be adapted from a combination of the tools that have been used in different policy auditing and analysis processes; the knowledge and experience of the research team on the subject; and from other similar studies conducted on the subject in different regions and with different purposes, addressing diet and physical activity as key NCD determinants and/or addressing NCD interventions in relation to adolescents [24,25,26]). The deductive framework will be applied across all study settings and adapted where necessary to suit the context in which the sub-studies will be conducted. Additional inductive themes from the coding of a ten percent sample of policy documents at each level will be incorporated into the coding framework and then applied to all included policy documents.

Based on the deductive and inductive themes, a common codebook will be developed to serve all six studies. Additional codes will be added for each study as relevant (the themes in the common codebook are outlined later).

We will then use a combination of discourse and content analysis as follows:

- First, we will describe each document, outlining the identifying features of each document (as summarized in Table 1).
- Second, a high-level description, drawing on the dimensions of the Walt and Gilson policy triangle, will be produced, depending on the level of detail provided in the documents. We will describe:
  ○ The context in which the document was created.
  ○ The process by which the document was produced, where this was evident in the documents.
  ○ The actors involved in the policy, captured either as individuals, or by organization and grouped by sector. This part of the analysis will allow us to ‘map’ all the actors that appear were identified in the documents as participating in the policy process (individuals, networks, agencies, etc.).

Next, we will conduct the content analysis, using a phased approach.

- First, we will provide a descriptive account of what the policy statements contain, for example, the exact phrases and terms used and the number of times important concepts appear (such as, for example how many times children or adolescent issues are addressed).
- Then, we will conduct a deeper discourse analysis, where we will assign interpretative meanings, overtly and possibly hidden, in the policy statements. We will interpret these statements within the contexts in which the policy documents were produced.
- A cross-code analysis will follow the detailed analysis of individual codes, where the research team for each sub-study will extract key analytical messages across codes. These will form the basis for reporting the results of each study and presenting them for peer review in a number of different formats (for example, peer review journal publications, policy briefs, policy roundtable discussions). The issues we will explore are outlined in Table 2.
- Finally, we plan to reflect on our findings from across the different sub-studies, particularly across the three studies at the country level, to identify similarities and differences between countries and identify leverages of support for diet and physical activity interventions where possible at the global, regional and country level.

The first level of analysis focused on the identifying and basic characteristics of each document (Table 1).

This descriptive information will guide the development of a policy timeline for each of the policy subsets (global to local).

We will further explore the extent to which policy positions that address similar issues are coherent, complementary, or contradictory, and we will identify any policy gaps. For example, we will examine the way documents address the issue of salt and sugar reduction and whether the policy statements situate this issue as an individual responsibility, or as a structural and collective societal responsibility. We will particularly interrogate how intersectoral strategies, and key actors and networks, appear to influence the nature of the policy content. Important convergences and/or contradictions across different sectors will be highlighted. For example, a trade and industry policy on sugar may conflict with a health policy intending to reduce sugar intake.

Overall, the key ‘outcome’ from the analyses will be to identify what the policy prescripts mean for; LMIC countries, with a focus on Africa, for intersectoral action, for adolescents and, if indicated in policies, for gender. Furthermore, in combination with the primary research collected on adolescents, families and stakeholders from different communities with social services such as education, and policy makers, will inform the next phase of the GDAR network, comprising the identification of key interventions for change.

#### 4.2.2. Privacy and Confidentiality

The research data from the policy analysis will be stored in electronic format for five years. The data will be shared with all members of the GDAR network, through a restricted access Team Drive on Google.

#### 4.2.3. Dissemination of Findings

The findings from the document review will be distributed in a variety of formats: As research reports and as presentations to other researchers and policy makers, particularly those based in the three selected countries and whom we will engage with for the development of interventions based on the findings (i.e., Kenya, Cameroon and South Africa); as policy briefs to make the information more accessible, in an easily-digestible format, to policy makers and other interested parties; a possible online African regional workshop, building on previous engagements with researchers and policymakers where the findings will be presented and discussed; as peer-reviewed articles for the global scientific community; as opinion pieces in popular media forums, policy briefs for the policy-making and practice audiences, in academic journals; and through presentations at relevant conferences linked to health policy and systems research.

## 5. Study Strengths and Limitations

### 5.1. Strengths

The strength of this study is that it involves researchers from different countries and with different levels of experience and insights. This study is further strengthened by the location of this work in a broader research network, rich in skill and expertise. The multi-level nature of the study means that policy on diet and physical activity, as important NCD determinants, could be examined from different vantage points, and in combination, will provide detailed insights into what global policy prescripts mean at a country level.

Further benefits derived from the study process, include the development of a rich repository of policy documents on an important public health issue, from multiple levels of the global system. In addition, study findings can potentially benefit country-level policy makers and practitioners, providing insight into the global and regional policies that influence and impact on local policy actions.

Furthermore, the study provides researchers involved in the broader GDAR network with available data, to compare and contrast policy positions across different country contexts and, as there is a growing interest in this area of work, it contributes to the knowledge base in this area.

The study team and the external advisors comprise a combination of experienced as well as early career researchers, where the early career researchers will be able to use a portion of the study in pursuit of higher-education degrees. This is true in at least four instances so far. The early career researchers are also now added to a relatively small group of health policy analysts, comprising a scare set of skills, in LMICs. Opportunities are also presented for early career researchers to attend professional NVivo coding and analysis training, contributing to skill development.

Identifying key sectors and actors allows researchers in this area to purposively identify important sectors with which to engage, and also which actors to approach for co-designing and co-producing NCD- and specifically diet and physical-activity related interventions.

### 5.2. Limitations

The limitations of the study are those commonly encountered in document analysis, in that the researcher’s ability to obtain the desired information from the policy document is confined to the detail provided in the document. Ideally, a policy document analysis should be combined with key informant interviews or engagement. The study team will compensate for this by planning a series of policy roundtable discussions where the results of the policy analysis are to be presented and discussed and, from there, to engage with policy makers and practitioners, locally and across LMICs in systematically developing interventions relevant to each context.

Importantly, as already indicated, this study is part of the broader GDAR network where other parts of the research have and will provide data obtained from engagements with a variety of key actors; the policy analysis is not a stand-alone piece of work, but integrally linked with research that will offer the further information in designing implementation interventions.

Finally, at a global level, one of the important sectors is the Trade and Industry sector. Due to limitations of time and expertise, this study will not engage with policies from the World Trade Organization (WTO). We have compensated for this by drawing on a review published in 2019 that examined the WHO response to the WTO member state challenges on tobacco, food and beverage policies, which highlighted important ways in which WHO-produced NCD policies were followed (or not) by Trade policies produced by the WTO [27]. We believe that the insights provided in the above-mentioned review will complement our insights.

## Figures and Tables

**Table 1:** Variables recorded for document identification.

Title
Year of publication
Country
Level at which document was produced (National, subnational, local)
Producing agency (Government department, non-Governmental agency, etc.)
Primary ‘ownership’ of the document, for example, the Department of Health, or Labour or WHO
Stated purpose of the document
Intended target audience(s) of the document
Intended timespan, if stated

**Table 2:** Cross-code content analysis-key analytical themes to be explored.

How is the problem of focus conceptualized in the policy documents?
How does this vary between issues and between sectors?
How are the target populations defined and described?
How are policy positions framed with respect to children and adolescents?
What values are embedded in the ideas of the policies about children and adolescents?
How are policy positions framed with respect to gender?
What values are embedded in the ideas of policies about gender?
How might these policy positions influence implementation?
Were deviations from global level policies evident in regional and country-level policies and how, by whom and why are these differences expressed?
How might these different meanings influence implementation, or explain/shape contestation among actors?
How do policies promote implementation (of which aspects and how)?
What are the ‘missing aspects’: missing actors, missing policy positions, missing sectors?

## Supplementary Materials

The following are available online at https://www.mdpi.com/article/10.3390/ijerph182413061/s1, File S1: Template letter when requesting policy documents, Table S1: Template for Codebook development.
