# Supplementary material for: Protocol for a Multi-Level Policy Analysis of Non-Communicable Disease Determinants of Diet and Physical Activity: Implications for Low- and Middle-Income Countries in Africa and the Caribbean
Source: Int J Environ Res Public Health. 2021 Dec 10;18(24):13061. doi: 10.3390/ijerph182413061 (PMC8700960; doi:10.3390/ijerph182413061)
Supplement: Supplementary file 1 [file ijerph-18-13061-s001.zip › File S1 Template letter when requesting policy documents.pdf]

SUPPLEMENTARY MATERIAL: TEMPLATE LETTER WHEN REQUESTING POLICY DOCUMENTS

Put on to your organisational letterhead

Amend letter as appropriate for your context and situation.

DATE:

Dear ……..

RE: REQUEST TO OBTAIN COPIES OF POLICY AND RELATED DOCUMENTS ON NON-COMMUNICABLE DISEASES AND INTERVENTIONS

Thank you for meeting with us/ agreeing to meet with us/talking/talk [SELECT APPROPRATE ONE] to us telephonically about the research we are doing on policies relating to non-communicable diseases and their determinants.

We are part of the GDAR network, which is a network of research partners in five different countries, doing research on various aspects of the determinants of non-communicable diseases. GDAR specifically focuses on Global Diet and Activity research and the research which we are doing in three African - and a Caribbean country, in partnership with Cambridge University in the United Kingdom, specifically aims to inform policy and interventions that would prevent and decrease the risk of non-communicable diseases. While we aim for our work to have global application, we are paying specific attention to communities and population groups that living in poverty and socio-economic disadvantaged circumstances. In this regard, we are hoping to work in partnership with our colleagues at various levels of government to jointly work towards evidence-based interventions that would decrease the rising prevalence of non-communicable diseases in our communities.

One of our starting points in GDAR is to come to grips with the available policies that are in operation at various levels of the system: global, national and subnational/local.

In [INSERT COUNTRY], we will be focusing on policies that are located at a national and a subnational [INSERT YOUR COUNTRY FOCUS] level. We are looking at policies that have been developed specifically for [INSERT COUNTRY] South Africa, as well as at policies that have been adopted from elsewhere, such as the World Health Organisation policy statements, guidelines and declarations, which a number of countries have adopted and then adapted for local application.

While a number of these policies are available on government and international agency websites, there are a few policy documents that may not be on the websites or that we have not yet identified and which you or others in your organisation are aware of. We will greatly appreciate your assistance in identifying and obtaining copies of such policies.

We will be doing a policy analysis on the available policies and identify the common policy goals; how these goals speak to one another across the different levels from global to local; as well as identify policy gaps that present the opportunity to further policy development at country level.

Our initial focus will be on policies that influence diet and also those that influence the availability of a healthy living environment, which will include the kind of environment that influences healthy physical activity.

We have obtained the necessary ethics permission for our policy analysis study and we enclose a copy of the ethics permission for your records.

We look forward to our next meeting/discussion with you, in which we will share with you the documents that we already have obtained, as well as the ones which we have identified but have not been able to get a copy from the available databases. This will give you a better idea of the ones that we might have missed or that we need to get a copy of.

Thank you once again for being so generous with your time and your assistance and we look forward to our future engagements with you. Please do not hesitate to let me know if you require any further information before we next meet.

Kind regards

[INSERT YOUR SIGNATURE AND RELEVANT CONTACT DETAILS]

Maylene Shung King

Associate Professor: Health Policy and Systems Division

Faculty of Health Sciences

University of Cape Town

Email: Maylene.shungking@uct.ac.za

Contact tel numbers: 021-4066580/071203607
